# User Experience of a Web-Based Mobile Health App Supporting Low-Income Pregnant Individuals With Diabetes: Mixed Methods Study

**DOI:** 10.2196/84061

**Published:** 2026-07-31

**Authors:** Sydney L Raucher, Layna Lu, Tazim Merchant, Elizabeth Soyemi, Charlotte Niznik, Rana Saber, Chen Yeh, Lynn M Yee

**Affiliations:** 1 Department of Obstetrics and Gynecology, Northwestern University Feinberg School of Medicine, 250 E. Superior Street, #5-2145, Chicago, IL, 60611, United States, 1 312-472-4685; 2 Research Application Design and Development Core Center, Northwestern University Feinberg School of Medicine, Chicago, IL, United States; 3 Department of Preventive Medicine, Biostatistical Collaboration Center, Northwestern University Feinberg School of Medicine, Chicago, IL, United States

**Keywords:** gestational diabetes mellitus, type 2 diabetes, mobile health, mHealth, pregnancy, usability, user experience, self-efficacy, social determinants of health

## Abstract

**Background:**

Diabetes mellitus management requires considerable patient self-efficacy, knowledge, and support for social determinants of health. These needs become particularly acute during pregnancy. Mobile health (mHealth) tools are a promising approach to enhance patient engagement with the health care system, education, and health promotion and may be particularly helpful during the period of rapid skills acquisition, which is a hallmark of experiencing diabetes during pregnancy. Therefore, we developed SweetMama, a web-based mHealth app designed to support and provide information to low-income pregnant individuals with gestational diabetes mellitus (GDM) or type 2 diabetes mellitus (T2DM).

**Objective:**

This study aimed to understand the user experiences of low-income pregnant people who were randomized to use SweetMama during a feasibility trial.

**Methods:**

This mixed methods secondary analysis of data from a feasibility randomized controlled trial (RCT) included participants randomized to SweetMama, an interactive, web-based mHealth app with multiple motivational and educational features that help reduce barriers to care, offer health education, and aim to improve diabetes self-care for low-income pregnant people. In the parent trial, English-speaking pregnant individuals with GDM or T2DM were randomized to use SweetMama during pregnancy or usual care. SweetMama users experienced an individualized curriculum from enrollment through 6 weeks postpartum. Upon exit, users completed 2 qualitative interviews (during the delivery hospitalization and at the postpartum visit) and surveys assessing standardized usability metrics. The surveys included the System Usability Scale (SUS), the Usefulness, Satisfaction, Ease of Use (USE) scale, and the mHealth App Usability Questionnaire (MAUQ) to assess usability. Qualitative data were analyzed using constant comparative techniques.

**Results:**

Of 30 SweetMama users, 60% (n=18) had GDM, 83.3% (n=25) had publicly funded prenatal care, and the majority identified as non-Hispanic Black (n=17, 56.7%) or Hispanic (n=11, 36.7%). Scores on the SUS (median 85.0/100, IQR 70.0‐88.8; ≥71% indicates acceptable or higher usability), USE (overall median 84.5/100, IQR 81.0‐91.4), and MAUQ (median 84.1/100, IQR 79.0‐91.3) indicated favorable usability assessments, particularly for the “ease of learning” domain. Qualitative interviews supported these findings: participants described the app as easy to navigate, well organized, and helpful for staying on track, citing features such as clear visual design, timely text reminders, and actionable tips. Users valued motivational elements and content specificity, while recommending increased customization and enhanced esthetics.

**Conclusions:**

In this user experience evaluation of a web-based mHealth app for low-income pregnant individuals with diabetes, participants found the tool to be user-friendly, visually appealing, informative, and motivating. Constructive feedback for application improvement for use in a future larger trial of clinical effectiveness was collected.

## Introduction

In the wake of a growing obesity epidemic, pregnant people are more likely to develop gestational diabetes mellitus (GDM) or enter pregnancy with type 2 diabetes mellitus (T2DM) [1-8]. Both GDM and T2DM increase the risk of maternal and fetal complications, such as preeclampsia, cesarean birth, and macrosomia, among others [3,6-10]. The rise in diabetes prevalence before and during pregnancy disproportionately affects low-income pregnant people of color, highlighting the need for interventions to incorporate social determinants of health in their development to reduce the risk of adverse outcomes [2,8-18].

Although diabetes treatment can lower the risk of complications [19-21], many logistical and social barriers prevent adequate diabetes self-management [22-27]. To optimize health outcomes, pregnant individuals with diabetes must be skilled in communication, organization, health literacy, and numeracy [22,25,28]. Strong social support systems are also beneficial [25,26,29-31]. Socially vulnerable individuals may not have sufficient access to educational resources for diabetes and are at greater risk of having suboptimal social support, leading to inadequate glucose control and poor health outcomes [25,26,28-31]. Thus, there is a need for interventions that educate patients, promote the skills needed for self-management, and address socioeconomic and racial disparities for pregnant individuals with diabetes [27,29].

Mobile health (mHealth) technology has the potential to address health disparities and promote health education through tailored and literacy-appropriate content [27]. With the widespread use of smartphones, mHealth can reach many individuals and reduce burdens such as transportation to and from health care providers [32]. In addition to being accessible, mHealth interventions are relatively low-cost and capable of providing support outside of clinical encounters [32,33]. mHealth tools for diabetes in nonpregnant populations have been effective at promoting self-management [34,35]. However, for both pregnant and nonpregnant populations, there is a lack of technology centered on increasing health literacy and self-efficacy skill development for diabetes management [36]. Furthermore, the tools that do exist are rarely designed to be user-centered (eg, they tend to rely on text message- or telemedicine-based communication rather than user-friendly applications) and are infrequently targeted toward individuals at greater social disadvantage [37].

To address the lack of mHealth tools aimed at supporting diabetes self-management among low-income, racially marginalized pregnant people, we created a multiphase project called SweetMama (NCT03240874). User feedback from an earlier phase of this project, which tested a text message–based intervention to promote diabetes self-management, expressed the desire for a more interactive, expansive mHealth tool [38]. In response, we developed the SweetMama app, a motivational and educational tool aimed at addressing diabetes self-management needs disproportionately experienced by English-speaking, socially vulnerable pregnant individuals [39]. Early feedback on this tool was explored in focus groups that included low-income, racially marginalized, English-speaking pregnant people. This feedback ensured that the app was literacy-appropriate and tailored toward the needs of this population (eg, by including preferred recipes and local resources). In the next phase of the study, a randomized controlled trial (RCT) was conducted to evaluate the feasibility of SweetMama use. The purpose of this planned secondary analysis from data gathered in the feasibility RCT is to evaluate the user experience of SweetMama participants, in order to consider how user experiences inform the optimization of mHealth interventions for this unique population.

## Methods

### Study Overview

**Figure 1.**
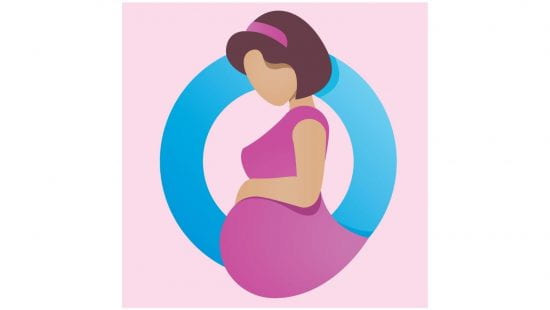
SweetMama logo.

**Figure 2.**
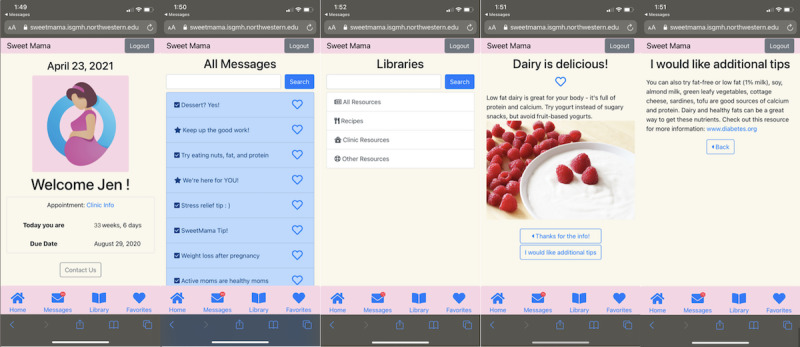
SweetMama screenshots of application features.

This is a mixed methods analysis of data collected from the SweetMama feasibility RCT, which aimed to understand the user experience with this web-based mHealth app, including usability and satisfaction. Per users’ requests from an earlier phase of the project, we created a web-based mHealth app, which expanded upon our previous text message–based intervention and was an accessible, interactive option for our patient population. The home screen of SweetMama provides the app’s logo as well as personalized information, including the participant’s name, gestational age, upcoming appointments, and a support portal (Figures 1 and 2). SweetMama delivered literacy-appropriate weekly motivational tips and individualized goal reminders, housed in a message center for easier access (Figure 2). Goal achievement was designed to include an element of gamification, whereby users could earn trophy icons for stating they had achieved their personalized goals. A library feature offered trusted educational materials, clinic-based resources, and recipes, with the option for users to “favorite” items for later use (Figure 2). If participants were interested in receiving further information from a certain message, they could click “I would like additional tips” to receive an in-depth description related to the message (Figure 2). During the postpartum period, the SweetMama curriculum focused on reminding participants about postpartum visit attendance, while also providing resources on diet maintenance during breastfeeding, mood changes, and more. This curriculum aimed to instill self-efficacy skills in patients and ensure that they were staying on track with the goals they set during pregnancy.

Data included in this analysis were generated from qualitative interviews addressing participants’ opinions and feedback on SweetMama, which was designed to provide insight into the app’s usability, as well as surveys assessing standardized usability metrics. The aim of the overarching SweetMama study was to test the feasibility and acceptability of this socially tailored, educational mHealth curriculum to encourage maternal self-efficacy for diabetes management among English-speaking pregnant individuals. This mixed methods secondary analysis analyzed user experiences and satisfaction with SweetMama, both of which were predesignated secondary outcomes of this study assessed only in the participants randomized to use SweetMama.

### Recruitment

In the parent trial, research team members met with SweetMama participants at 3 separate visits: the enrollment visit (<30 wk of gestation), delivery visit, and postpartum visit. Consent was obtained at the enrollment visit. Individuals were recruited from the study site, Northwestern Medicine, at the initiation of prenatal diabetes care. Participants met the following inclusion criteria: age 18 and older, GDM or T2DM, English-speaking, confirmed intrauterine pregnancy prior to 30 weeks’ gestational age, low income (defined as use of public insurance for prenatal care or household income <200% of the poverty line for family size), and access to a smartphone. This analysis focuses only on data from those who were randomized to use SweetMama. Users engaged with SweetMama throughout their pregnancy.

### Quantitative Data Collection and Analysis

Users completed 3 validated, standardized assessments of usability at the time of their delivery hospitalization or within the first week after delivery. These included the System Usability Scale (SUS), the Usefulness, Satisfaction, Ease of Use (USE) scale, and the mHealth App Usability Questionnaire (MAUQ). The SUS, USE, and MAUQ were scored using standard scoring strategies for each scale, with scores normalized to a 0 to 100 scale for each questionnaire. The SUS survey is a 10-item questionnaire using a 5-point Likert scale that provides a measure of overall user satisfaction [40,41]. A system is “acceptable” if scores are above 70, “marginal” if scores are between 51 and 70, and “not acceptable” if scores are 50 and below [41]. The USE survey (a 30-item questionnaire), which similarly assesses the usability of a system through a 7-point Likert scale, has components that address 4 domains: usefulness, ease of use, ease of learning, and satisfaction [40,42]. The MAUQ (21 items, 7-point Likert scale) assesses ease of use and satisfaction, system information arrangement, and usefulness in its version specifically for interactive mHealth tools and patients [43]. These tests are also reliable indicators of usability, acceptance, and satisfaction for evaluating mHealth resources (Cronbach α=0.91 [SUS], 0.98 [USE], 0.90 [MAUQ]) [41-43]. All scores were converted to a standard scale of 0 to 100. Descriptive summary statistics (median and IQR) were calculated. Quantitative analyses were performed using Stata v15 (StataCorp).

### Qualitative Data Collection and Analysis

Two interviews were conducted to gather qualitative data about participants’ experiences. The first interview occurred during the participants’ delivery hospitalization or within the first week after delivery. Research personnel used an interview guide developed by the research team to ask questions and obtain feedback on areas of improvement for SweetMama through addressing three domains: (1) user experience, (2) app functionality (including features, curriculum, and preferences), and (3) user diabetes management. All interviews were conducted by a trained full-time research team member who was not involved in participants’ clinical care, and who had no prior clinical relationship with participants. Participants were informed that their responses would not affect their medical care or study incentives, and they were encouraged to provide both positive and negative feedback to improve SweetMama. The second interview took place at the postpartum visit, approximately 6‐8 weeks postpartum. This qualitative interview focused on SweetMama’s postpartum curriculum, with questions tailored toward feasibility, improvement, and user engagement with the app. Interviews lasted approximately 20 to 40 minutes. They were audio-recorded and transcribed using a professional service. Qualitative data were analyzed using the constant comparative method with a team-based approach [44,45]. A subset of 5 transcripts was reviewed by trained independent coders, who identified recurring concepts, referred to as subthemes, related to users’ experiences with SweetMama. These subthemes were used to develop a preliminary codebook, which was discussed and refined by the full research team to ensure clarity and consistency. Two independent coders trained using Dedoose (SocioCultural Research Consultants, LLC), a qualitative coding software, then used the revised codebook to iteratively code the transcripts. The codebook continued to be updated during regular team meetings, and those changes were applied. Any discrepancies between coders were resolved on a code-by-code basis. Delivery and postpartum interviews were analyzed as a single corpus because the interview guides were designed to capture complementary aspects of a continuous user experience over time. Thematic saturation was determined through an iterative, team-based process.

### Mixed Methods Integration

We used a mixed methods design in which qualitative interviews and quantitative usability surveys were collected during the same study visits and analyzed independently. Following separate analyses, results were brought together during interpretation using a narrative, side-by-side approach to examine areas of convergence and divergence. Quantitative usability scores were used to contextualize qualitative themes, and areas of alignment or mismatch were discussed during team meetings. These integrated insights informed overall conclusions on the user experience of SweetMama.

### Ethical Considerations

All participants provided written informed consent prior to enrollment. Participants received up to US $100 via gift cards for their participation in the parent trial (US $25 for the enrollment visit, US $50 for the delivery visit, and US $25 for the postpartum visit). Participants who completed an interview received an additional US $10. This study was approved by the Northwestern University Institutional Review Board (IRB STU00205609).

## Results

### Overview

From January 2020 to October 2020, 30 participants were randomized to use SweetMama and were included in this analysis. Of these 30 participants, 18 had GDM and 12 had T2DM. The majority identified as non-Hispanic Black (n=17, 56.7%) or Hispanic (n=11, 36.7%). The majority (n=21, 70%) had completed at least some college education. All participants met the study criteria for low-income status, and 83.3% (n=25) had publicly funded prenatal care (Table 1).

**Table 1.** Participant demographics of SweetMama.

Characteristic	SweetMama users (N=30)
Age (y), mean (SD)	31.4 (4.7)
Self-reported race and ethnicity, n (%)	
Non-Hispanic white	1 (3.3)
Hispanic	11 (36.7)
Non-Hispanic Black	17 (56.7)
American Indian/Alaska Native	1 (3.3)
Medicaid, n (%)	25 (83.3)
Education, n (%)	
Some high school or less	3 (10)
High school graduate	6 (20)
Some college or technical school	9 (30)
College or technical school graduate	12 (40)
Relationship status, n (%)	
Married	14 (46.7)
Single or other relationship status	16 (53.3)
Nulliparous, n (%)	6 (20)
Current work status, n (%)	
Work full-time	11 (36.7)
Work part-time	8 (26.7)
Unemployed, student, or other employment status	11 (36.7)
Body mass index at first prenatal visit, kg/m^2^ (n=29), mean (SD)	42.94 (12.4)
Diabetes diagnosis during pregnancy, n (%)	
Gestational diabetes mellitus	18 (60)
Type 2 diabetes mellitus	12 (40)
Gestational age at enrollment >20 weeks, n (%)	14 (46.7)

### Quantitative Usability Metrics

At the delivery visit, 28 participants completed the surveys, and 2 participants were lost to follow-up. SUS, USE, and MAUQ scores demonstrated positive experiences with the SweetMama tool (Table 2). The median score on the SUS survey was 85.0/100 (IQR 77.0‐88.8), and 71.4% (n=20) of participants found SweetMama to be acceptable. This generally positive feedback was corroborated by similarly high USE scores (median score 84.5/100, IQR 81.0‐91.4), with the 4 domains of the USE scale (usefulness, ease of use, ease of learning, and satisfaction) scoring 82.1 (IQR 71.4‐85.7), 88.3 (IQR 84.4‐94.8), 91.1 (IQR 85.7‐100.0), and 82.7 (IQR 73.5‐93.9), respectively. Finally, the median MAUQ score was 84.1/100 (IQR 79.0‐91.3). All quantitative metrics suggest favorable usability assessments.

**Table 2.** System Usability Scale (SUS), Usefulness, Satisfaction, Ease of Use (USE) scale, and mHealth App Usability Questionnaire (MAUQ) quantitative metrics (N=28).

Scale and score type	Values
SUS	
Overall score, median (IQR)	85.0 (70.0‐88.8)
Acceptability, n (%)	
Acceptable (>70)	20 (71.4)
Marginal (51-70)	7 (25)
Not acceptable (<51)	1 (3.6)
USE scale, median (IQR)	
Overall score	84.5 (81.0‐91.4)
Usefulness score	82.1 (71.4‐85.7)
Ease of use score	88.3 (84.4‐94.8)
Ease of learning score	91.1 (85.7‐100.0)
Satisfaction score	82.7 (73.5‐93.9)
MAUQ, median (IQR)	
Overall score	84.1 (79.0‐91.3)

### Qualitative User Experience Data

#### Qualitative Themes

At the delivery visit, 28 participants completed interviews, while 2 participants were lost to follow-up. At the postpartum visit, an additional 3 participants were lost to follow-up, resulting in 25 completed postpartum interviews. Most participant feedback centered on technical aspects of the application that enhanced the user experience. Analysis revealed four major themes; participants cited aspects of the (1) usability, (2) visual features, (3) content, and (4) frequency of communication in their feedback. Themes and subthemes, as well as participant-identified areas for improvement, are described later and in Table 3.

**Table 3.** Qualitative interview quotes on SweetMama user experience.

Theme and subtheme	Exemplary quotation
Usability	
Ease of use	“The app, it was easy, self-explanatory. You really didn’t need anybody to explain it to you. But it was helpful in the sense that you know where to go and what you were doing, if that makes sense.”
Organization	“Everything was set up in folders. I remember that. Motivation, goals. It was well organized. Easy to go back into and see what I wanted to remember or to read again.”
Visual features	
Logo	“Well, I love the picture of the pregnant mommy. But mainly it, it comes directly to my, to my messages, so I was able to see when I had one from you guys. And they come, it came right up, so it was like, you know if, even if I didn’t open it up right away, I could just click on the link and then still get it sent back to me. You know…that was cool.”
Gamification	“I mean it had recipes and notes and little trophy award things if you were doing stuff correctly. It’s like you had your own personal champion cheering you on. So yeah [I liked it].”
Content	
Tips and messages	“I think the tip messages were slightly more specific and I appreciated that. It gave me something more specific of a goal or an idea to think of just like when it’s like did you sign up for WIC or this is a way to like relieve stress specifically or this is a specific way to increase your protein and your snacking and things like that. I felt like it was very tangible.”
Goals	“It’s still giving me information and still staying on top of what I need to be doing. So if I need to set a goal and you guys do remind me to stay on that goal…”
Recipes	“I really did like that because I know things that I thought were okay to eat, I would go on there and I would look at it and it was high in sugars so it was kind of new to me and I was like okay, I didn’t know that.”
Appointment reminders	“There were appointments that I would completely forget about and your text messages actually helped.”
Frequency of communication	
Appropriate frequency	“[Message frequency] was just right to me…to keep you active…So you know how you get an app or something and you don’t even use it you know. So like keep you active.”

#### Usability

Themes emerged relating to two specific aspects of the app’s usability: (1) ease of use and (2) app organization. SweetMama was widely appreciated for its *ease of use* by the cohort. When asked about their experience with the app, participants found SweetMama to be self-explanatory and easy to navigate. SweetMama also helped promote feelings of self-efficacy, as participants felt in control of their app experience. One participant commented: “You really didn’t need anybody to explain it to you.”

The *organization* of the app also helped participants with the comprehension of resources. It was easy for users to find the specific information they were seeking and to return to information they had saved or “favorited” for later review. One participant reported: “The goals are where the goals are… it’s easy to get the stuff you need, information you need. You know it’s easy.”

#### Visual Features

SweetMama users favored the visual features of the app. These attributes helped promote user engagement, as they created a sense of recognition and reward when accessing and using the application. Two specific areas of positive feedback regarding visual recognition were the logo or icon and gamification aspects.

Regarding the *logo*, participants appreciated the SweetMama logo of the pregnant mother. Participants felt like they were able to relate to the picture: “It shows me that it, you know that it’s about expecting mothers.”

The use of a representative logo created a sentiment of belonging and support. Participants also reported that the logo was recognizable and aided in identifying information from the application.

Participants also enjoyed the addition of awards to the app. *Gamification* heightened participants’ sense of accomplishment and motivation to continue striving to achieve their goals: “It’s like you had your own personal champion cheering you on.”

#### Content

Another highlight of the SweetMama app was its content. Analysis revealed that the feedback centered around four key areas: (1) tips and messages, (2) goals, (3) recipes, and (4) appointment reminders.

First, *tips and messages* served as a guiding force, coaching participants on what they were “supposed to do.” Participants liked how holistic the tips and messages were, targeting “every aspect of diabetes,” and were even excited to see new content from SweetMama: “I was curious about what was the tip or what was the goal this time, you know? I was always looking forward to reading the messages.”

In addition, participants noted that the specificity of the messages and the tangible nature of the tips made lifestyle goals more realistic to accomplish. Participants liked the references to specific foods to incorporate into their diets or support programs to find locally or online.

*Goals* set in the app also helped participants stay on track with diabetes management. Multiple participants reported that the goals motivated them to “work a little bit harder.” Several participants reported how the goal-setting function and the reminders about those goals specifically helped with being proactive and motivating action: “They were inspirational, they made me want to use the app more… They made me want to set goals and complete them and then go back into the app and show that I actually did something.”

Many participants reported diet as their largest struggle. Thus, they found *recipes* on SweetMama especially useful to help them navigate their new diets. Participants appreciated that SweetMama gave them guidance on diet, which helped dispel any uncertainties: “things that I thought were okay to eat, I would go on [SweetMama] and I would look at it and it was high in sugars...and I was like okay, I didn’t know that.” Recipes addressed issue areas such as portion sizes and the blood sugar levels of foods. Participants also appreciated the variety in recipe options, which covered meals and snacks.

Finally, the SweetMama text messaging system for *appointment reminders* was effective in helping participants remember their appointments. One participant shared: “There were appointments that I would completely forget about and your text messages actually helped.”

#### Frequency of Communication

Participants were specifically queried about the frequency of communication and reminders within the SweetMama curriculum. Most participants found that the communication was of an appropriate intensity (3 times per wk, with additional reminders for upcoming appointments). The frequency promoted user engagement, retained user activity, and did not detract from their purpose.

#### Areas for Improvement

In addition to strengths, participants identified several areas for improvement. Interview guides included explicit prompts asking users to describe frustrations, dislikes, and suggested changes to encourage candid negative feedback. Suggested improvements clustered into 3 primary themes.

First, participants desired greater personalization, including the ability to tailor goals, reminders, and educational content to their specific diabetes type, treatment plan, and daily routines.

Second, users noted that the app’s visual design and esthetics felt “rudimentary” and recommended enhanced graphics, more engaging layouts, and opportunities for customization (eg, personalized icons or themes) to improve user engagement.

Third, participants expressed interest in expanded content breadth, such as additional practical resources and more individualized messaging throughout pregnancy and postpartum. These findings highlight opportunities to refine SweetMama’s usability in future iterations.

## Discussion

### Principal Findings

This study used both qualitative and quantitative methods to examine the user experience of a novel web-based mHealth app designed to augment health literacy and patient motivation and reduce logistical and social barriers for marginalized English-speaking pregnant individuals with diabetes. To our knowledge, this is among the first mixed methods evaluations of a pregnancy-focused, web-based mHealth app specifically designed to be literacy-conscious, responsive to practical barriers (eg, time constraints and food access), and intended to support individuals experiencing socioeconomic challenges. SweetMama users demonstrated high usability, including high satisfaction scores, based on quantitative assessments completed during postpartum hospitalization. These favorable usability metrics were corroborated by qualitative data; in interviews, participants cited usability, visual features, content, and frequency of communication as beneficial. Participants perceived appointment reminders and goal-tracking features as helpful for navigating competing responsibilities and time-management challenges common in settings of socioeconomic stress or low social support. Specific, actionable tips, including guidance on WIC enrollment (Women, Infant, Children—a federally funded nutrition program implemented by the Chicago Department of Public Health), affordable nutrition strategies, and recipes, were described as useful by participants facing food insecurity and limited nutrition literacy. Participants also valued the app’s timely messages, motivational elements, and gamification, which contributed to perceived encouragement and support. Together, these findings illustrate how SweetMama’s design features function as practical interventions for social barriers to diabetes self-management in an English-speaking, low-income pregnant population. In terms of areas of improvement, participants expressed a desire for increased personalization and esthetic appeal of the application, such as tailoring the tool’s goals to patients’ specific diabetes needs and allowing users to personalize the icon.

### Comparison to Previous Work

These findings are consistent with the literature on mHealth interventions for patients with GDM. While various mHealth options for GDM have been developed, only a few have evaluated the user experience of their participants. Nicholson et al [46] developed a web-based intervention, GooDMomS, which was designed to change the behavior of and educate users with GDM through informative web lessons, an online self-monitoring diary (for glucose, exercise, and diet tracking), recipes, a peer-support message board, and weekly text messages. Similar to our findings, guidance on diet was an important component for these participants. One new feature that users in GooDMomS desired was a list of suggested entrées from restaurants, rather than solely providing recipe options [46]. Personalization was also underscored in GDM literature as an important feature of an mHealth app. Duan et al [32] found that participants desired customizable features for diet and exercise goals so that they would be able to receive information about foods and workouts that were amenable to them. This individualization can help to engage patients and make them feel as though the tool is addressing their specific needs [32]. Our findings expand on existing literature by providing a mixed methods approach among a low-income, racially and ethnically diverse population of urban, American pregnant people.

These themes were also reflected in the broader type 1 diabetes mellitus (T1DM) and T2DM literature, in which several mHealth interventions have been evaluated for nonpregnant populations. In an mHealth app called Diabetes Journey, Schmidt et al [47] implemented a learner-centered curriculum for adolescents with T1DM. Consistent with our findings, the researchers found that the following factors promoted usability: interactive components, consistency in content and interaction design, variety, and interface esthetics. Furthermore, in a qualitative evidence synthesis of T2DM mHealth tools, O’Neill et al [48] found that people living with T2DM felt motivated to use mHealth tools for self-management. Similar to our findings, they favored applications that are visually appealing and incorporate features to communicate with a provider [48].

Existing literature also noted features that can be considered or expanded upon in future iterations of SweetMama. Participants in the study by Duan et al [32] emphasized that amidst the increased stress during pregnancy, being able to quickly reach out to a health care professional to ask questions would help relieve uncertainty and reduce the burden of traveling to the clinic. SweetMama did not include direct, personalized patient-to-provider communication options as patients had access to the institution’s electronic medical record patient portal for such communication. Since participants were advised to use that portal for clinician communication, interviews did not directly query about this concept, but future work may benefit from incorporating a connection to the portal within an mHealth educational tool. Additionally, while participants did not explicitly mention peer support as a desired feature of SweetMama, this could serve as another aid in diabetes self-management, as users could interact with other people with similar experiences and goals [49-51]. Peer support can foster a sense of community and emotional support among pregnant individuals, which can be especially helpful for SweetMama’s target audience who may lack adequate psychosocial support. Future work may aim to identify ways to connect users through a virtual SweetMama community of active users.

### Strengths and Limitations

This study’s strengths lie both in its population and its mixed methods approach. SweetMama was tailored specifically toward socially vulnerable people with fewer socioeconomic resources. Historically, the health concerns of socially vulnerable populations have been neglected; this study adds to the existing literature by obtaining feedback from a more diverse population regarding an emerging web-based mHealth app focused on building self-efficacy, motivation, and health literacy. These perspectives are key in building equitable mHealth interventions and can inform the development of future iterations of the tool. An additional strength is the study’s use of both standardized surveys and interviews; the SUS assessed the overall usability of SweetMama, USE assessed user satisfaction and ease of learning, and MAUQ was specifically tailored to mHealth tools. When coupled with qualitative interviews and rigorous methodological analysis, we were able to glean a holistic understanding of the SweetMama user experience.

However, this study has several limitations. First, participants were recruited from a single urban academic institution and represent a relatively narrow demographic scope, which limits the generalizability of findings to pregnant people receiving care in other geographic, clinical, or socioeconomic settings. The small sample size and single recruitment location, both of which were appropriate for this phase of work, may not capture the full range of experiences among individuals with GDM or other high-risk pregnancies. For this phase of work, our sample size aligned with established norms for usability testing and mixed methods evaluations, allowed us to achieve thematic saturation in qualitative interviews, and was sufficient to detect patterns in quantitative usability scores, which showed limited variation across participants. Nonetheless, the sample lacked representation from Asian and other underrepresented groups. Additionally, all participants had to be English-speaking, despite diabetes being prevalent in Hispanic and immigrant populations, due to budgetary limitations during the intervention development process [1,2]. Future work must include a larger sample size with increased racial and ethnic representation to more fully develop and test an intervention tailored to different cultures. Another necessary next step is translating the app into different languages to suit a more linguistically diverse patient population, as well as studying the effect of SweetMama use on clinical outcomes.

The visual appeal of the app was also limited due to budgetary constraints; therefore, future work toward greater customization and enhanced graphics could enhance the appearance of SweetMama. Next, while we did not formally integrate datasets (eg, by comparing qualitative responses with quantitative scores), the qualitative and quantitative findings were used to complement one another and provide a more holistic understanding of user experiences. Due to limited variation in user scores (ie, scores were generally high), further subgroup analysis was not feasible. In addition, because participants had regular contact with the research team and received incentives for study completion, social desirability bias may have influenced both usability ratings and qualitative feedback. Finally, the current study was designed to evaluate perceived usability, satisfaction, and early user experiences with SweetMama; sustained user engagement and clinical effectiveness were beyond its scope and should be assessed in future studies.

### Future Directions

Based on our findings, several key design implications for maternal mHealth tools emerge: (1) prioritize ease of learning and low-burden access to support engagement among users with competing demands; (2) incorporate structured, organized content with actionable guidance (eg, recipes, nutrition tips, and resource navigation) to address health literacy and social needs; (3) include reminders and goal-tracking features to facilitate self-management and appointment adherence; and (4) leverage motivational framing, timely messaging, and light gamification to enhance user engagement and perceived social support. With these in mind, there are several clinical implications that can be gleaned from this work. mHealth apps like SweetMama can be highly appealing to users if they incorporate visual and knowledge-building features, while remaining focused on basic usability concepts such as ease of use and ease of learning. Applications designed with patient-centered features may support initial uptake and user satisfaction. These factors are ultimately important for sustained user engagement and improved self-management, particularly for those with greater social needs. Although SweetMama is not yet available outside of the research context, the lessons learned from this assessment can inform the development and sustainability of other interactive mHealth tools to support pregnant patients with diabetes. Our findings suggest that future maternal mHealth interventions may benefit from emphasizing low-burden interaction, actionable content, supportive reminders, and motivational features that help users integrate self-management tasks into their daily routines. For example, features such as appointment reminders, gamification, and a diverse array of recipe options, educational tips, and messaging are recommended by participants for inclusion in future designs. Additionally, new iterations of SweetMama can be created in different languages to support wider audiences and reduce health literacy and language barriers for non–English-speaking individuals. Future work may further consider whether and to what extent the experiences of users with different types of diabetes (eg, diet-treated vs medication-treated; pregestational vs gestational) may warrant alterations of interventions such as SweetMama. Further personalization can include opportunities to tailor the application based on stage of pregnancy or postpartum status, dietary preferences and restrictions, reminder frequency, and desired support resources.

In conclusion, mHealth tools are a promising avenue for GDM self-management, education, and addressing disparities. SweetMama users found this web-based mHealth app to be engaging and motivational, with its strengths lying in usability, visual features, and content. Areas for improvement included increased customization of the app’s features, such as the logo and personal diabetes needs. These findings support user satisfaction and the perceived usefulness of the tool, but further research is needed to evaluate sustained engagement with the app, behavior change, and clinical outcomes. Future directions for this work also include testing SweetMama in a larger sample size, in multiple languages, and across multiple institutions. In addition, implementing new features, such as peer support and connection to patient portals, may enhance the user experience.

## Abbreviations

GDM: gestational diabetes mellitus
MAUQ: mHealth App Usability Questionnaire
mHealth: mobile health
RCT: randomized controlled trial
SUS: System Usability Scale
T1DM: type 1 diabetes mellitus
T2DM: type 2 diabetes mellitus
USE: Usefulness, Satisfaction, Ease of Use
WIC: Women, Infant, Children

## References

[1] Pu J, Zhao B, Wang EJ (2015). Racial/ethnic differences in gestational diabetes prevalence and contribution of common risk factors. Paediatr Perinat Epidemiol.

[2] Peng TY, Ehrlich SF, Crites Y (2017). Trends and racial and ethnic disparities in the prevalence of pregestational type 1 and type 2 diabetes in Northern California: 1996-2014. Am J Obstet Gynecol.

[3] Catalano PM (2010). The impact of gestational diabetes and maternal obesity on the mother and her offspring. J Dev Orig Health Dis.

[4] Shah NS, Wang MC, Freaney PM (2021). Trends in gestational diabetes at first live birth by race and ethnicity in the US, 2011-2019. JAMA.

[5] Zhou T, Du S, Sun D (2022). Prevalence and trends in gestational diabetes mellitus among women in the United States, 2006-2017: a population-based study. Front Endocrinol (Lausanne).

[6] Huet J, Beucher G, Rod A, Morello R, Dreyfus M (2018). Joint impact of gestational diabetes and obesity on perinatal outcomes. J Gynecol Obstet Hum Reprod.

[7] Alexopoulos AS, Blair R, Peters AL (2019). Management of preexisting diabetes in pregnancy: a review. JAMA.

[8] Sridhar SB, Ferrara A, Ehrlich SF, Brown SD, Hedderson MM (2013). Risk of large-for-gestational-age newborns in women with gestational diabetes by race and ethnicity and body mass index categories. Obstet Gynecol.

[9] American College of Obstetricians and Gynecologists Committee on Practice Bulletins—Obstetrics (2018). ACOG Practice Bulletin No. 201: pregestational diabetes mellitus. Obstet Gynecol.

[10] Guo H, Zhang Y, Li P, Zhou P, Chen LM, Li SY (2019). Evaluating the effects of mobile health intervention on weight management, glycemic control and pregnancy outcomes in patients with gestational diabetes mellitus. J Endocrinol Invest.

[11] Diaz-Santana MV, O’Brien KM, Park YMM, Sandler DP, Weinberg CR (2022). Persistence of risk for type 2 diabetes after gestational diabetes mellitus. Diabetes Care.

[12] Guariguata L, Linnenkamp U, Beagley J, Whiting DR, Cho NH (2014). Global estimates of the prevalence of hyperglycaemia in pregnancy. Diabetes Res Clin Pract.

[13] Schwartz N, Nachum Z, Green MS (2015). The prevalence of gestational diabetes mellitus recurrence--effect of ethnicity and parity: a metaanalysis. Am J Obstet Gynecol.

[14] Bower JK, Butler BN, Bose-Brill S, Kue J, Wassel CL (2019). Racial/ethnic differences in diabetes screening and hyperglycemia among US women after gestational diabetes. Prev Chronic Dis.

[15] Bellamy L, Casas JP, Hingorani AD, Williams D (2009). Type 2 diabetes mellitus after gestational diabetes: a systematic review and meta-analysis. Lancet.

[16] Egan AM, Enninga EAL, Alrahmani L, Weaver AL, Sarras MP, Ruano R (2021). Recurrent gestational diabetes mellitus: a narrative review and single-center experience. J Clin Med.

[17] Venkatesh KK, Lynch CD, Powe CE (2022). Risk of adverse pregnancy outcomes among pregnant individuals with gestational diabetes by race and ethnicity in the United States, 2014-2020. JAMA.

[18] Daneshmand SS, Stortz S, Morrisey R, Faksh A (2019). Bridging gaps and understanding disparities in gestational diabetes mellitus to improve perinatal outcomes. Diabetes Spectr.

[19] Poolsup N, Suksomboon N, Amin M (2014). Effect of treatment of gestational diabetes mellitus: a systematic review and meta-analysis. PLoS One.

[20] Hartling L, Dryden DM, Guthrie A, Muise M, Vandermeer B, Donovan L (2013). Benefits and harms of treating gestational diabetes mellitus: a systematic review and meta-analysis for the U.S. Preventive Services Task Force and the National Institutes of Health Office of Medical Applications of Research. Ann Intern Med.

[21] Lende M, Rijhsinghani A (2020). Gestational diabetes: overview with emphasis on medical management. Int J Environ Res Public Health.

[22] Yee LM, McGuire JM, Taylor SM, Niznik CM, Simon MA (2016). Factors promoting diabetes self-care among low-income, minority pregnant women. J Perinatol.

[23] Yee LM, McGuire JM, Taylor SM, Niznik CM, Simon MA (2015). “I was tired of all the sticking and poking”: identifying barriers to diabetes self-care among low-income pregnant women. J Health Care Poor Underserved.

[24] Yee LM, McGuire JM, Taylor SM, Niznik CM, Simon MA (2016). Social and environmental barriers to nutrition therapy for diabetes management among underserved pregnant women: a qualitative analysis. J Nutr Educ Behav.

[25] Nielsen KK, Kapur A, Damm P, de Courten M, Bygbjerg IC (2014). From screening to postpartum follow-up - the determinants and barriers for gestational diabetes mellitus (GDM) services, a systematic review. BMC Pregnancy Childbirth.

[26] Carolan M (2013). Women’s experiences of gestational diabetes self-management: a qualitative study. Midwifery.

[27] Anderson-Lewis C, Darville G, Mercado RE, Howell S, Di Maggio S (2018). mHealth technology use and implications in historically underserved and minority populations in the United States: systematic literature review. JMIR mHealth uHealth.

[28] Pirdehghan A, Eslahchi M, Esna-Ashari F, Borzouei S (2020). Health literacy and diabetes control in pregnant women. J Family Med Prim Care.

[29] Muhwava LS, Murphy K, Zarowsky C, Levitt N (2020). Perspectives on the psychological and emotional burden of having gestational diabetes amongst low-income women in Cape Town, South Africa. BMC Womens Health.

[30] Rad GS, Bakht LA, Feizi A, Mohebi S (2013). Importance of social support in diabetes care. J Educ Health Promot.

[31] Miller TA, Dimatteo MR (2013). Importance of family/social support and impact on adherence to diabetic therapy. Diabetes Metab Syndr Obes.

[32] Duan B, Liu Z, Liu W, Gou B (2022). Assessing the views and needs of people at high risk of gestational diabetes mellitus for the development of mobile health apps: descriptive qualitative study. JMIR Form Res.

[33] Carrandi A, Hu Y, Karger S (2023). Systematic review on the cost and cost-effectiveness of mHealth interventions supporting women during pregnancy. Women Birth.

[34] Kitsiou S, Paré G, Jaana M, Gerber B (2017). Effectiveness of mHealth interventions for patients with diabetes: an overview of systematic reviews. PLoS One.

[35] Al-Hashmi I, Hodge F, Nandy K, Thomas E, Brecht ML (2018). The effect of a self-efficacy-enhancing intervention on perceived self-efficacy and actual adherence to healthy behaviours among women with gestational diabetes mellitus. Sultan Qaboos Univ Med J.

[36] Alqudah A, McMullan P, Todd A (2019). Service evaluation of diabetes management during pregnancy in a regional maternity hospital: potential scope for increased self-management and remote patient monitoring through mHealth solutions. BMC Health Serv Res.

[37] Zahmatkeshan M, Zakerabasali S, Farjam M, Gholampour Y, Seraji M, Yazdani A (2021). The use of mobile health interventions for gestational diabetes mellitus: a descriptive literature review. J Med Life.

[38] Yee LM, Leziak K, Jackson J (2021). Patient and provider perspectives on a novel mobile health intervention for low-income pregnant women with gestational or type 2 diabetes mellitus. J Diabetes Sci Technol.

[39] Yee LM, Leziak K, Jackson J (2023). SweetMama: usability assessment of a novel mobile application among low-income pregnant people to assist with diabetes management and support. Diabetes Spectr.

[40] Hajesmaeel-Gohari S, Khordastan F, Fatehi F, Samzadeh H, Bahaadinbeigy K (2022). The most used questionnaires for evaluating satisfaction, usability, acceptance, and quality outcomes of mobile health. BMC Med Inform Decis Mak.

[41] Lewis JR (2018). The system usability scale: past, present, and future. Int J Human Comput Inter.

[42] Gao M, Kortum P, Oswald F (2018). Psychometric evaluation of the USE (Usefulness, Satisfaction, and Ease of USE) questionnaire for reliability and validity. Proc Hum Factors Ergon Soc Annu Meet.

[43] Zhou L, Bao J, Setiawan IMA, Saptono A, Parmanto B (2019). The mHealth app usability questionnaire (MAUQ): development and validation study. JMIR mHealth uHealth.

[44] Glaser BG (1965). The constant comparative method of qualitative analysis. Soc Probl.

[45] Hewitt-Taylor J (2001). Use of constant comparative analysis in qualitative research. Nurs Stand.

[46] Nicholson WK, Beckham AJ, Hatley K (2016). The Gestational Diabetes Management System (GooDMomS): development, feasibility and lessons learned from a patient-informed, web-based pregnancy and postpartum lifestyle intervention. BMC Pregnancy Childbirth.

[47] Schmidt M, Lu J, Luo W (2022). Learning experience design of an mHealth self-management intervention for adolescents with type 1 diabetes. Educ Technol Res Dev.

[48] O’Neill M, Houghton C, Crilly G, Dowling M (2022). A qualitative evidence synthesis of users’ experience of mobile health applications in the self-management of type 2 diabetes. Chronic Illn.

[49] Alexandra Friedman M, Niznik CM, Bolden JR, Yee LM (2016). Reciprocal peer support for post-partum patients with diabetes: a needs assessment for the Diabetes Buddy Program. J Community Health.

[50] Safiee L, Rough DJ, Whitford H (2022). Barriers to and facilitators of using eHealth to support gestational diabetes mellitus self-management: systematic literature review of perceptions of health care professionals and women with gestational diabetes mellitus. J Med Internet Res.

[51] Ingstrup MS, Wozniak LA, Mathe N (2019). Women’s experience with peer counselling and social support during a lifestyle intervention among women with a previous gestational diabetes pregnancy. Health Psychol Behav Med.

